# Comparative Study of Volatile Compounds in the Fruit of Two Banana Cultivars at Different Ripening Stages

**DOI:** 10.3390/molecules23102456

**Published:** 2018-09-25

**Authors:** Xiaoyang Zhu, Qiumian Li, Jun Li, Jun Luo, Weixin Chen, Xueping Li

**Affiliations:** State Key Laboratory for Conservation and Utilization of Subtropical Agro-Bioresources/Guangdong Provincial Key Laboratory of Postharvest Science of Fruits and Vegetables, College of Horticulture, South China Agricultural University, Guangzhou 510642, China; xiaoyang_zhu@scau.edu.cn (X.Z.); lqm209@126.com (Q.L.); leegun@scau.edu.cn (J.L.); luojun125335.cool@163.com (J.L.); wxchen@scau.edu.cn (W.C.)

**Keywords:** banana cultivars, ripening physiology, ethylene, fruit quality, volatile compounds, esters

## Abstract

Aromatic compounds are important for fruit quality and can vary among fruit cultivars. Volatile compounds formed during the ripening of two banana cultivars, Brazilian and Fenjiao, were determined using headspace solid-phase micro-extraction (SPME) and gas chromatography coupled with mass spectrometry (GC-MS). These two cultivars exhibited different physiological characteristics during storage. Fenjiao fruit exhibited faster yellowing and softening, a higher respiration rate and greater ethylene production. Also, the soluble sugar content in Fenjiao fruit was much higher than in Brazilian fruit. In total, 62 and 59 volatile compounds were detected in Fenjiao and Brazilian fruits, respectively. The predominant volatile components isoamyl acetate, butanoic acid, 3-methyl-3-methylbutyl ester, hexanal, *trans*-2-hexenal and 1-hexanol varied during ripening stages. Moreover, esters were more abundant in Fenjiao, and propanoic acid 2-methylbutyl ester, and octanoic acid were only detected in Fenjiao. These compounds contribute to the unique flavors and aromas of the two cultivars.

## 1. Introduction

Banana is one of the most important crops in tropical and sub-tropical areas [1]. Banana is also the second largest fruit crop, and China is one of the three biggest producers in the world [1,2]. Brazilian (*Musa* spp. AAA group) is a banana cultivar widely cultivated in China due to its high productivity, large fruit and good long-term storage properties, but it is susceptible to diseases, and peel-browning. Fenjiao (*Musa* ABB Pisang Awak) is more popular because of its good flavor and abiotic stress resistance. It is also widely cultivated in many areas in China [3]. Fenjiao fruit have good taste, high sugar content, rich nutrition, and a unique and pleasant aroma, which is different from other cultivars. However, Fenjiao fruit has a short shelf-life after harvest, which leads to serious deterioration in fruit quality [4].

Aroma is an important indicator of fruit flavor and an important characteristic of fruit. It can be used to distinguish different types of fruits or different fruit cultivars [5,6]. Fruit aromas are particularly sensitive to compositional alterations and can be altered by a wide range of pre-harvest or postharvest conditions [7,8]. For instance, banana fruit-flavor quality is affected by a series of ripening processes. The factors that influence fruit flavor and the possible mechanisms have been reviewed, including genotype, cultural practice, the stages of ripening, and storage conditions [9]. Studies on aromatic compounds provide chemical information about the fruit aroma composition, the in vivo aroma biosynthetic pathway, the accumulation and evolution of aromatic compounds, as well as providing helpful guidance for plant breeding, fruit processing, crop storage and regulation of aromatic compounds [6,10].

The aromatic properties of banana are important for its attractiveness as a fresh fruit. Banana fruit has a pleasant flavor and is widely consumed throughout the world. The typical flavor compounds of banana are produced during a short ripening period, and its aroma composition fluctuates during different stages, forming unique aroma signatures [11]. Several studies on the aroma compositions of banana during ripening have been reported, but most focused on the chemical compositions of the aroma at the mature period or during fruit processing [11,12]. The changes in aroma composition at different fruit-ripening stages have also been reported [13], which are helpful to understand the formation of aromatic compounds during fruit ripening. The predominant volatile components of different banana cultivars vary, which gives them their own unique flavor. For example, the volatile component 3-methylbutyl butanoate is the predominant ester of the Cavendish cultivar [14]. In general, esters give a fruity note aroma [7]; for example, isoamyl butanoate, 3-methylbutyl acetate and isoamyl isovalerate contribute to the banana’s fruity odor [15,16]. It was reported that 246 volatile compounds have been identified in banana fruit, including 112 esters, 57 alcohols, 39 acids, 10 aldehydes, and 10 ketones, but only 12 compounds contribute significantly to banana aroma [16].

The Fenjiao (*Musa* ABB, group) banana variety has been widely consumed throughout the world and is becoming more popular due to its nutritional value and pleasant flavor [17]. Most studies on the aromatic components of fresh banana fruit have focused only on the *Musa* AAA cultivars, and little is known about the aroma components for Fenjiao [13,16]. In this work, we showed that Fenjiao and Brazilian banana fruit have significantly different aromatic profiles during fruit ripening. The objectives of this study were to characterize the aromatic profiles of Brazilian and Fenjiao during the fruit ripening process using solid-phase microextraction (SPME) coupled with gas chromatography-mass spectrometry (GC-MS) and to compare the differences in the volatile components at different stages of ripening. The results obtained in this work facilitate our understanding of the main aromatic compounds affecting fruit quality in Fenjiao and Brazilian banana fruit.

## 2. Results

### 2.1. Physiological Characteristics of Ripening Brazilian and Fenjiao Banana Fruit

**Figure 1:**
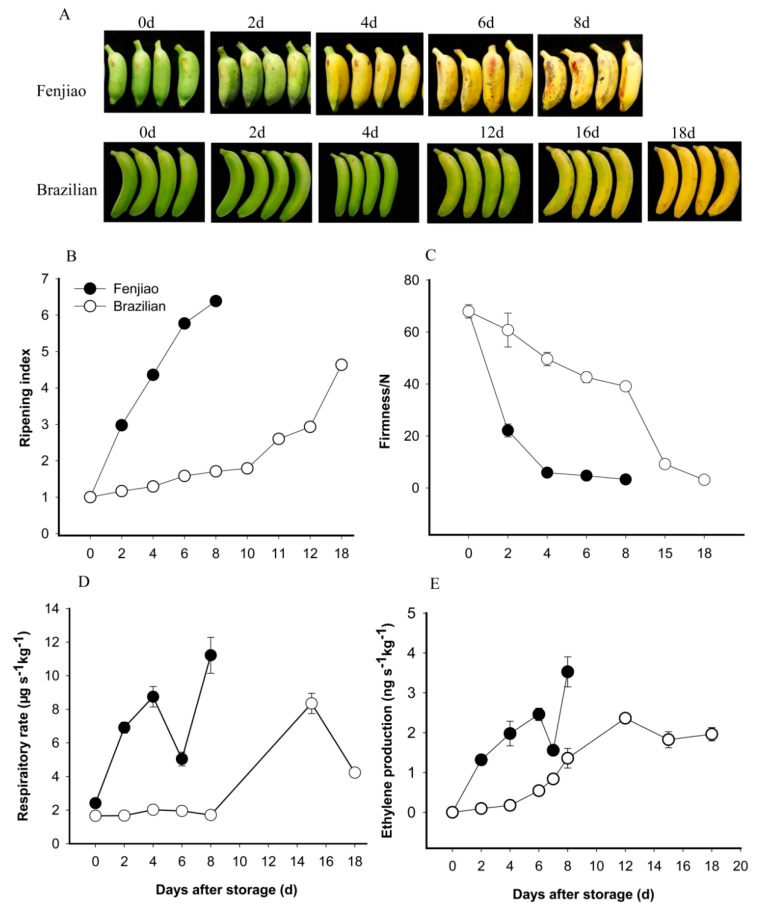
The ripening profiles of Brazilian and Fenjiao banana fruit. (**A**) Pictures of Fenjiao and Brazilian banana fruit during storage at room temperature (22 ± 1 °C); (**B**) fruit ripening index; (**C**) fruit firmness; (**D**) fruit respiration rate; and (**E**) ethylene production. The values presented were obtained from three independent biological replicates. The bars indicated the standard errors (mean ± SE).

A change in peel color is visible during fruit ripening. As shown in Figure 1A, the Fenjiao banana ripening was very fast at room temperature. The color of the fruit peel changed from green to yellow by the 6th day post-harvest, which indicates that fruit were fully ripened. However, for Brazilian banana fruit, the changes in fruit peel color were much slower. Fruit slowly started to change color on the 10th day post-harvest and reached the full ripening stage on the 18th day. The storage period for Brazilian was about 20 days. Fenjiao banana fruit ripened much more rapidly than the Brazilian fruit.

Fruit firmness is another important index for fruit ripening. As shown in Figure 1B, fruit firmness decreased during storage. Different banana cultivars showed a distinctly different downward trend. Fenjiao fruit firmness declined rapidly on the 2nd day from 70 N to 20 N and then gradually declined to a low level of 3 N (Figure 1B). However, for Brazilian fruit, firmness declined slowly during the early stage and then declined more rapidly during the later period. Firmness reached a low level on the 15th day (Figure 1B).

Banana is a typical climacteric fruit. As shown in Figure 1C, the respiration rate of Fenjiao fruit increased rapidly and reached a significant respiration peak on day 4, followed by a decline. Fruit respiration increased again on day 6 and reached to another peak on day 8, which may be due to the fruit deterioration (Figure 1C). For Brazilian fruit, the respiration rate remained at a steady low level during the first 8 days and then increased significantly after day 8, reaching a peak on the 15th day (Figure 1C).

Ethylene production of Fenjiao fruit showed similar trends to that of the respiration rate. As shown in Figure 1D, ethylene production of Fenjiao fruit increased rapidly and reached a peak on the 6th day. Ethylene production began to decline on the 6th day and reached a second peak on the 8th day. For Brazilian banana fruit, ethylene production increased gradually and reached a maximum on the 12th day. The maximum ethylene production was observed on the 6th day and the 15th day for Fenjiao and Brazilian banana fruits, respectively (Figure 1D).

### 2.2. Changes of Soluble Sugar Content at Different Maturation Stages Post-Harvest

The changes in the contents of glucose, fructose and sucrose, the three main sugar components in banana fruit, were determined during fruit ripening. As shown in Table 1, the contents of these sugars were quite low during the early stage post-harvest. The sugar contents increased rapidly with fruit ripening. For Fenjiao banana fruit, the content of glucose increased from 29.2 mg·g^−1^ to 112.5 mg·g^−1^, and fructose content increased from 33.3 mg·g^−1^ to 99.3 mg·g^−1^. Sucrose content significantly increased and then declined when the fruit was fully ripened on day 6. Similar changes in sugar content were observed for Brazilian banana fruit. However, the sugar contents were significant lower than that of Fenjiao fruit at the mature-green stage. All the sugar components in Brazilian banana fruit were significantly lower than those of Fenjiao fruit during the entire storage period, except for the content of sucrose at the fully ripened stage (Table 1).

### 2.3. Volatile Compounds of Banana Fruit (Musa AAA Group, Brazilian) at Different Stages of Ripening

As shown in Table 2, in Brazilian banana fruit, 62 compounds, including esters, alcohols, aldehydes, ketones, acids and other minor compounds, were identified at three stages of ripening using the SPME method. The predominant volatile compound was aldehyde at the stage of mature-green, which accounted for 69.31% of the total content, while compounds such as hexanal and *trans*-2-hexenal accounted for 35.92% and 31.17%, respectively. It has been reported that the C_5_, C_6_-alcohol, aldehyde and ketone together form a grassy aroma [18]. These aldehydes have grass typical flavor smells, which represented the characteristic aroma substances of banana at this stage. In addition, the level of 1-hexanol was quite high, accounting for 21.47% of the total content. However, few esters were detected at this stage.

At the color-turning stage, the levels of ′green aroma′ compounds decreased dramatically, and a large number of esters were generated. Acetate, butyrate and methyl-butyrate ester were the most abundant esters, accounting for more than 70% of the total esters produced. 1-butanol, 3-methyl-, acetate (14.34%), butanoic acid, 3-methylbutyl ester (18.7%), and butanoic acid, 3-methyl-, 3-methylbutyl ester (14.03%) were also present. These esters are the most important compounds at the ripening stage of banana fruit. Among them, the branched-chain ester accounted for 85.49% of the total esters. In addition, some hydrocarbons were detected at this stage, and the aldehyde composition did not change significantly.

When fruit reached the full-ripening stage, total volatile production increased continuously and slowly. The contents of certain typical aroma compounds such as 1-butanol, 3-methyl-, acetate (from 14.34% to 11.55%), butanoic acid, 3-methyl-, 3-methylbutyl ester (from 14.03% to 13.71%) remained relatively stable at this stage, but butanoic acid, 3-methylbutyl ester decreased from 18.7% to 11.9%. The proportion of total aldehydes decreased rapidly. Meanwhile, the level of ethanol increased. The variety and relative contents of ketones increased gradually, but hydrocarbons were not detected at this stage.

### 2.4. Volatile Compounds of Banana Fruit (Musa ABB Group, Fenjiao) at Different Stages of Ripening

As shown in Table 3, 59 compounds were identified in banana fruit (*Musa* ABB group, Fenjiao), including esters, alcohols, aldehydes, ketones, acids and other minor compounds. Again, the most abundant volatile compounds were aldehydes at the stage of mature-green, accounting for the total content of 58.8%. C_6_-aldehydes were the predominant aldehydes, such as hexanal (22.5%), *trans*-2-hexenal (25.08%) and 2-hexenal (11.16%). In addition, the relative content of 1-hexanol was high, which contributed to the total volatile content of 21.12%. Few esters were detected at the mature-green stage, and the results for the Brazilian banana were similar.

At the yellowing stage, the contents of esters and alcohols increased gradually. Meanwhile, aldehyde contents decreased slowly. At this stage, fruit aroma content remained relatively low, and the characteristic compounds had not yet formed.

When Fenjiao fruit were fully ripened, large amounts of esters were generated, accounting for 90.01% of the total volatile compounds produced. Butanoic acid, 1-methyl, hexyl ester reached to 21.79%, 1-butanol, 3-methyl-, acetate reached to 21.29%, and butanoic acid, 3-methyl-, 3-methylbutyl ester, hexyl acetate, and 3-methylbutyl isobutyrate reached 6.24%, 6.11%, and 5.71%, respectively. Among these, the branched-chain ester accounted for 79.68% of the total esters. Meanwhile, the proportion of total aldehydes and alcohols decreased rapidly. 1-hexanol was not detected at this stage, but other alcohols appeared (Table 3).

In addition, during the storage period for the Fenjiao banana, the composition and relative contents of hydrocarbons decreased steadily, and hydrocarbon acids were present at the later stage, which may be converted from the earlier hydrocarbon species.

### 2.5. Comparison of the Main Volatile Compounds between Brazilian and Fenjiao Banana Fruits at Different Stages

The major volatile components of Brazilian and Fenjiao fruits were identified and compared at three different ripening stages (Table 4). At the mature-green stage, about 70% of the total volatiles consisted of C_6_-aldehydes and C_6_-alcohols. The unsaturated aldehydes represented the typical aroma of mature-green banana fruit, and this result is in agreement with a study by Zhu et al. [13].

Esters are the main aromatic compounds during the yellowing of banana fruit. As the fruit ripened, the ester compounds in Fenjiao fruit increased slow firstly and then increased rapidly. While in banana cultivar Brazilian, the ester contents increased rapidly at first and then increased slowly. However, in general, the relative concentrations of ester compounds increased consistently as the fruit ripened. There were no significant differences in the content of esters between ripened fruits (90.01% and 73.43%, respectively). There were high concentrations of acetate, butyrate, methyl butyrate and other methyl acetates in Brazilian cultivar, which accounted for the most abundant aromatic compounds. Nevertheless, the Fenjiao is rich in acetate, butyrate and contained small amounts of methyl-(acetate, propionate) butyrate. Some other esters, such as octoate and decanoate, were detected in Fenjiao cultivar but were not found in the Brazilian banana fruit.

The main alcohol substance in Brazilian banana was 1-hexanol, and its relative content was reduced gradually during fruit ripening. In addition to 1-hexanol, other alcohols were also present in Fenjiao banana.

### 2.6. PCA Analysis at Different Ripening Stage of Two Banana Varieties

**Figure 2:**
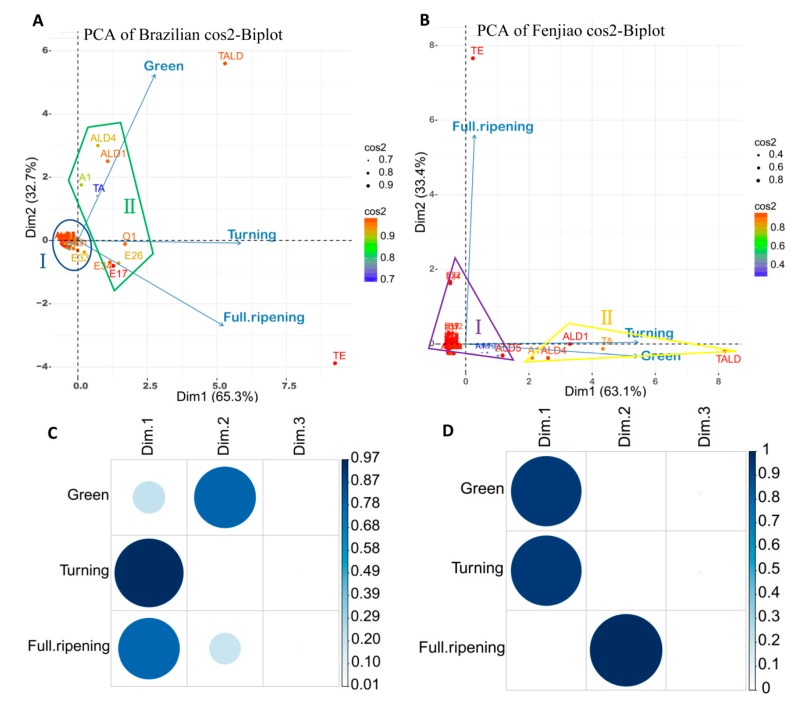
Overview of volatiles profiles of two banana varieties. (**A**,**B**) Biplot of PCA analysis described in the banana varieties (Brazilian and Fenjiao) of all variables during the three fruit ripening stages. (**C**,**D**) The cos2 of volatiles compounds at three ripening stages on all the dimensions for Brazilian and Fenjiao, respectively. Different volatiles were characterized by PCA, and the code in (**A**,**B**) were listed in Supplemental Table S1. Dim: Direction, which indicated the principal component (PC). PCA analysis was conducted and visualized using R software. The cos2 of variables on all the dimensions using the corrplot package. A high cos2 indicates a good representation of the variable on the principal component.

Principal component analysis (PCA) was performed to provide a general overview of the samples and an easy visualization of the complete data set in a reduced dimension plot. It was also used to get a primary evaluation of maturity stage of samples, to explore interdependencies among the tested volatiles and to identify variant volatiles groups with similar characters per cultivar. For this analysis, all volatile production at three ripening stage were studied in this work. As shown in Figure 2, volatiles production showed quite different characters in the Fenjiao and Brazilian banana fruit.

For the cv. Brazilian (Figure 2A), the first two components could explain 98% of the variability among the samples, i.e., 65.3% (principal component 1, PC1) and 32.7% (principal component 1, PC1). Generally, volatiles components were distributed from the positive to the negative side on the *x*-axis (PC1) according to different ripening stages (Figure 2A). Different distribution indicates different maturity stages of the samples. As we can see that green stage situated in the upper and full-ripening stage in the lower quadrant. And turning stage located in the middle, which on the *x*-axis. Aldehydes are the most important volatile component for the green stage of Brazilian banana fruit, as the total aldehydes contribute most to the green stage. ALD1 (*trans*-2-hexenal) and ALD4 (hexenal) are the principal, main contributors to the total aldehyde content. For the fruit at turning stage, esters, aldehydes and others non-name volatiles were important. E17 (butanoic acid, 3-methyl-, 3-methylbutyl ester), E26 (butanoic acid, 3-methylbutyl ester), E34 (1-butanol, 3-methyl-, acetate) and O1 (others volatiles) are the principal, main contributors to the volatiles at this stage. Esters were the predominance of volatiles at the full ripening stage, which the total esters contribute most to the volatiles at full ripening stage. E17 (butanoic acid, 3-methyl-, 3-methylbutyl ester), E26 (butanoic acid, 3-methylbutyl ester and E34 (1-butanol, 3-methyl-, acetate) are the most produced ester at this stage.

The k-means clustering analysis showed that two main group were distinguished for the volatiles production in Brazilian fruit. One group situated in the origin of the coordinate, which including most of the esters and adehydes components. Volatiles in this group were mainly produced at the fruit turning and full ripening stage (Figure 2A). The second group located in the middle part of the coordinate, including *trans*-2-hexenal, hexanal and 1-hexanol etc., which accumulated during both stage of green and turning stage.

For the cv. Fenjiao (Figure 2B), the first two components could explain 96.5% of the variability among the samples, i.e., 63.1% (PC1) and 33.4% (PC1). As shown in Figure 2B, Fenjiao banana fruit showed quite different volatiles characters from Brazilian fruit. The volatiles at green and turning stage grouped together, which both situated on the *x*-axis. Aldehydes and alcohols were the predominance of volatiles at the turning and green stage for Fenjiao fruit, which mainly presented the PC1. It was significantly different from Brazilian fruit, which esters and aldehydes were mainly presented the PC1. They are grouped together and represent the volatiles at the green and turning stages. ALD1 (*trans*-2-hexenal), ALD4 (hexanal), ALD5 (2-hexenal) and A1 (1-hexanol) are the principal, main contributors to the volatiles at the green and turning stages. Most of the esters grouped together and situated in the origin of the coordinate. Esters were the predominance of volatiles of Fenjiao fruit at full ripening stages, which the total ester almost located on the y-axis, with the same direction of full ripening stage. Among them, E22 (butanoic acid, 1-methyl, hexyl ester) and E34 (1-butanol, 3-methyl-, acetate) are the main contributors to the esters at this stages (Figure 2B).

Cos2 values in Figure 2C,D showed the representation quality of sample features on factors map. A high cos2 indicates a good representation of the variable on the principal component. Brazilian and Fenjiao fruit showed quite different volatiles characters at different ripening stage. For Brazilian fruit, volatiles at green stage mainly contribute to the PC2, and had little contribution to PC1. Volatiles at turning and full ripening stage perfectly represented by the PC1, which mainly presented by esters. However, for Fenjiao fruit, volatiles at green and turning stage were well represented by the PC1, excluding from other PCs. And volatiles at full ripening stage were perfectly represented by the PC2, excluding from PC1 (Figure 2C,D).

## 3. Discussion

Different varieties and cultivars of banana fruits may be different in their storage capacity and flavors due to the differences in their physiological characteristics (weight, size, shape, texture and color), physicochemical properties (pH, titratable acidity, soluble solids, moisture content, and total solids), chemical properties (soluble sugars, vitamin C, starch, pectic substances, and volatile compounds) and sensory attributes (appearance, flavor, odor, color, firmness) [16,19]. For example, the genome of B group (B-*Musa balbisiana*) cultivars showed a greater resistance to low-temperature stress compared to the AAA group (A-*Musa acuminata*) [20]. Different banana cultivars produce different volatile compounds in response to cold storage, enhancing their ability to tolerate low temperatures [21]. It has also been reported that growth altitude affects the composition of volatile compounds [7].

Two cultivars of banana, Brazilian and Fenjiao, were selected for this study. Our results showed that Fenjiao fruit ripened much more rapidly than the Brazilian fruit (Figure 1). Fenjiao fruit turned yellow more rapidly than Brazilian fruit, and fruit firmness decreased rapidly for Fenjiao. The respiration rate and ethylene production were much greater in Fenjiao banana fruit compared to the Brazilian fruit (Figure 1D,E). These results indicate that the storage capacity of Fenjiao banana fruit is much shorter than Brazilian fruit, which may mainly be due to the high respiration rate, highly commercial harvested maturation, genotype, peel structure, etc. Respiration is an excellent indicator of metabolic rate of fruit, and it can also work as a useful criteria for the storability of fresh produce [22]. Normally, horticulture produce with a low respiration rate (such as apple, potato, carrot, etc.) show long-term storability while high respiring produce (such as strawberry, papaya, banana and litchi) have a short storage life. Fenjiao fruit showed high respiration rate than Brazilian, which well explained that Fenjiao fruit showed short storage life. Ethylene is well known as a ripening hormone, which can trigger ripening in climacteric fruits and senescence in non-climacteric fruits, vegetables and ornamental plants [23]. High ethylene production also accelerate fruit ripening. Fenjiao fruit also showed high ethylene production than Brazilian and with a short storage time. Manipulating ethylene production is an effective way to either promote rapid and predictable ripening of climacteric fruits or to delay ripening. The postharvest technologies of controlling fruit respiration and ethylene have been extensively studied [24]. As an ethylene receptor inhibitor and a nontoxic antagonist of ethylene, 1-methylcyclopropene (1-MCP) has been employed to increase the shelf life of various climacteric and non-climacteric fruits, which can effectively prolong the shelf-life of both Fenjiao and Brazilian banana fruit. Our previous work showed that the combination of 1-MCP and ethylene could effectively delay banana fruit ripening [24].

Glucose, fructose and sucrose are three main sugars in banana fruit during fruit ripening [25]. Actually, the abundance of all three sugars was significantly higher in Fenjiao fruit than in Brazilian fruit (Table 1). The contents of the three sugars in the fruit of the banana increased with fruit ripening and reached a maximum at full ripening. Our results show that the most abundant sugars in Fenjiao banana are glucose and fructose, which is different from most fruits, such as litchi, pineapple, and other banana cultivars, in which sucrose is the predominant sugar at full ripening [26]. In the Brazilian banana fruit, when the fruit reached full maturity, the contents of three kinds of sugar were similar to one another, and the Brazilian contained higher concentrations of sucrose but lower concentrations of glucose and fructose than Fenjiao fruit. The content of total soluble sugar in Fenjiao banana at each period was higher than that of Brazilian banana fruit, indicating that Fenjiao banana fruit are much sweeter than Brazilian banana fruit. Flavor results from the complex interaction of taste (sweetness, sourness) and aroma. Taste is basically determined by sugars and acids [27]. Sugar and acid levels are the main factors affecting fruit flavor acceptability, and high sugar content indicate the sweetness [27]. The most important taste components in banana affecting sweetness are glucose, fructose, and sucrose [12]. Sugar accumulation (along with acids) may also determine aroma intensity [28]. In tomato, it was found that cultivars with the increasing in sugar level enhanced the aroma intensity and made the overall flavor more acceptable. Indeed, sugars are positively correlated with overall flavor acceptability [28]. Ethylene also play an important role in the sugar metabolism. A recent work showed that ethylene affects sugar metabolism in climacteric and non-climacteric plums [29]. Ethylene reduced sucrose catabolism and induced sucrose biosynthesis in different plum types. In the present work, high ethylene production in Fenjiao fruit than Brazilian fruit was observed, corresponding to high sugars content detected in Fenjiao fruit than Brazilian fruit.

Production of aromatic volatile compounds is important to the flavor of banana fruit and is the defining characteristics of different cultivars. The characteristic banana aroma is not derived from just one or a few volatile compounds but is the result of a complex mixture of volatile compounds [30]. Generally, esters provide fruity notes and are responsible for the characteristic aroma of fresh banana fruit. Esters constitute the major class of compounds present in the volatile profile of banana fruit, such as butyl acetate, isoamyl acetate, ethyl acetate, butyl butanoate and isoamyl isobutanoate [21,31].

The volatile compounds of many banana cultivars have been widely studied, but this is the first report on the aromatic profiles of preferable banana cultivars Fenjiao and Brazilian [21]. Headspace SPME coupled with GC-MS provides a simple, rapid and reliable technique to analyze volatile compounds in banana fruit. In the present work, the volatile compounds of Fenjiao banana fruit and Brazilian banana fruit were determined during different ripening stages. Our results show that 62 and 59 volatile compounds, including esters, alcohols, aldehydes, ketones, hydrocarbons and acids, were detected in the two banana cultivars, respectively. The most abundant aromatic compounds in the two banana cultivars during ripening were isoamyl acetate, butanoic acid, 3-methyl-3-methylbutyl ester, hexanal, *trans*-2-hexenal and 1-hexanol. Differences in the volatiles composition of the two banana varieties and significant variations within the same banana variety were observed at different stages of ripening (Supplementary Figures S1 and S2). However, nine compounds, including acetate, caproate, butyrate, valerate, methyl acetate, methyl propionate, methyl butyrate, C_6_-aldehydes and C_6_-alcohols were shared. In general, as the fruits ripened, the concentrations of unsaturated aldehydes and alcohols decreased, while the concentrations of esters increased significantly.

It has been proposed that esters are the main aromatic compounds in banana fruit (*Musa* AAA group) [15]. Zhu et al. determined that acetate, butyrate and 3-methyl butyrate are the most abundant esters in fully ripened banana fruit. Isoamyl acetate represented the characteristic compound of banana fruit. However, other reports found that butyrate and propionate are the main esters in banana fruit (*Musa* AAA group) [12], and the levels of 2-hexenal, hexyl acetate, butanoic acid, 3-methyl-3-methylbutyl ester, butanoic acid, 3-methylbutyl ester and hexanoic acid 3-methylbutyl ester are very high [12,18]. Our results are similar to those reported in previous studies, except hexyl acetate was not detected in our study, and the content of hexanoic acid 3-methylbutyl ester was low, which may be due to many factors, such as different varieties, cultivation conditions, and post-harvest storage conditions [7]. Moreover, different origins of banana varieties have different aromatic compositions and concentrations [14,21]. Previous work showed that hexyl acetate was the main esters in banana samples (cv. Gran Enano, *Musa* spp., AAA-genome group), which is originated from three different locations from Central and South America (Costa Rica, Panama and Colombia) [12], and its volatiles production was significantly affected by ethylene treatment. However, for other cultivars such as cv. Nanicão (*M. acuminata*, AAA, harvested from São Paulo State, Brazil) and cv. Prata (*M. acuminata* X M. balbisiana, AAB, harvested from Minas Gerais State, Brazil), hexyl acetate was not detected or at very low level, and the volatiles vary between those two cultivars and are greatly affected by storage temperature [21]. Similarly, our previous work could not detect or only detected very low level of hexyl acetate in Brazilian banana fruit [13], but high hexyl acetate content was detected in Fenjiao fruit in the present work.

Our data also show that the predominant volatile compounds in Fenjiao banana were similar to those of the Brazilian banana at the mature-green stage, such as C_6_-aldehydes, C_6_-alcohols, and abundant esters, especially isoamyl acetate, at the fully ripened stage. The main esters in Fenjiao banana were acetate, butyrate, methyl acetate, methyl propionate and methyl butyrate, which were different from Brazilian banana. Except for isoamyl acetate, including butanoic acid, 1-methylhexyl ester, hexyl acetate, butanoic acid, 3-methyl-3-methylbutyl ester and propionic acid, 3-methyl-3-methylbutyl ester contents were very high, and they were the main volatile compounds in Fenjiao banana. Some propionate and octanoate esters were generated during the later ripening stage in the Fenjiao fruit, which also contribute to the fragrance of hybrid strawberry [32]. Hexyl acetate is also a characteristic aromatic compound of apple fruit [33]. Therefore, the aroma of Fenjiao banana is similar to that of apple and strawberry. During the later fruit ripening stage, the Fenjiao fruit produced propanoic acid, 2-methylbutyl ester, octanoic acid, 3-methyl butyl ester and 3-methylbutyl decanoate, which form a special kind of aroma.

Ethylene plays an important role in the ripening of climacteric fruits by regulating an array of ripening-associated processes and the biosynthesis of aromatic compounds [34]. It has been shown that ethylene is important in the production of aromatic volatiles in some climacteric fruit, such as banana [35], apple [34], tomato [36] and sweet melon [37]. Otherwise, its inhibitory effect on ethylene biosynthesis in these fruits can reduce the production of aromatic compounds [37]. It was reported that in apple, the metabolism of volatile compounds is ethylene-dependent, and ethylene significantly enhanced volatiles production, whereas 1-MCP (1-methylcyclopropene, a nontoxic antagonist of ethylene) treatment inhibited volatiles [34]. In apple, the expression of most of the genes involved in the biosynthesis of aromatic compounds is regulated in an ethylene-dependent manner. In citrus fruit, which are considered non-climacteric fruit, ethylene affects the production of aromatic compounds in a variety-specific manner [8].

Volatile esters are generated mainly by esterification of alcohols and acyl-CoA derived from both fatty acid and amino acid metabolism [38]. Fatty acids serve as ester precursors and are metabolized through two major pathways: β-oxidation and the lipoxygenase (LOX) system [39]. LOX may play a major role in the formation of straight-chain volatile compounds in fruits [40]. The enzyme LOX catalyzes the hydroperoxidation of polyunsaturated fatty acids. These products are further metabolized into aldehydes, alcohols and volatile esters. The decomposed unsaturated fatty acids form C6-aldehydes and C_6_-ethanols [10]. Branched-chain volatile compounds contribute to the aroma of many fruits and are derived from the metabolism of branched-chain amino acids. Branched-chain amino acids are converted into branched-chain alcohols, which are coupled with acyl-CoA, forming branched-chain esters [41]. In these reactions, the transformation from acids and alcohols to esters could be catalyzed directly by alcohol acyltransferases (AATs). The aldehydes are transformed into acids or alcohols [42]. AAT catalyzes the esterification reaction between alcohols and acyl-CoA and is directly responsible for the generation of volatile esters [43]. Furthermore, alcohol dehydrogenases (ADHs) participate in the transformations between aldehydes and alcohols. *AAT* gene expression in apple is reported to be ethylene-dependent, but ADH and LOX seem to be independent of ethylene regulation [44]. Our previous work showed that the expression of *MaHPL*, *MaLOX*, *MaAAT*, *MaADH* and *MaPDC* were closely related to the volatiles production in Brazilian banana fruit [45]. Actually, the biosynthetic pathways related to aromatic volatiles were controlled and regulated by different genes, which form a complex network [10]. Therefore, the observed changes in the production of volatile compounds between Fenjiao and Brazilian banana might be due to different metabolic pathways, modifications in the regulation and functional properties of precursor-generating enzyme activities as well as the regulation of corresponding genes. More specifically, the availability of substrate for the biosynthesis of volatile esters is believed to be a limiting step in ester production [46]. However, the mechanisms for the volatiles production difference and volatiles biosynthesis key factors between Fenjiao and Brazilian banana fruit are not clear so far. Further works are needed to interpret the underlying mechanism of these difference.

## 4. Materials and Methods

### 4.1. Plant Materials

Two cultivars of mature, green banana fruit (*Musa* AAA group, Brazilian and *Musa* ABB group, Fenjiao) were obtained from an orchard in Panyu (Guangzhou, China). All fruits were cleaned and soaked in 0.2% (*w*/*v*) chlorine cleaner solution for 10 min to eliminate potential microbes. The fruits exhibited no visual symptoms of any disease or blemishes and were uniform in weight, shape, and maturity. The fruits were stored at 22 ± 1 °C for natural maturation. Three independent measurements were conducted on randomly selected fruit as replicates for each group of fruit at the mature-green, turning, and full-ripening stages, respectively. For Fenjiao fruit, sampling were conducted on 0, 2 and 6 days after harvest, which corresponded to mature-green, turning, and full-ripening stages. For Brazilian fruit, sampling were conducted on 0, 12 and 18 days after harvest, which corresponded to mature-green, turning, and full-ripening stages.

### 4.2. Fruit Ripening Evaluations

The fruit ripening process was evaluated and monitored by periodical measurements of different physical indices, including fruit respiration, ethylene, firmness and visual inspection of the peel color. Fruit color was evaluate by the fruit ripening index, which is on a scale from 1 to 7 as described by Zhu et al. [13]. Fruit respiration, ethylene production and fruit firmness were determined as described by Zhu et al. [24].

### 4.3. Changes in Sugar Content in Banana Fruit during Fruit Ripening

Glucose, fructose and sucrose are the main sugar components in banana fruit [25]. Ion chromatography was used to determine sugar components in banana fruit during the ripening process, as described by Wang et al. [47], with minor modification. Briefly, 1 g of banana pulp and 50 mL of distilled water were crushed by a cell grinder (BILON96-II, Shanghai, China), and the sugars were extracted 60 times by ultrasonic oscillation (ultrasonic time of 3 s, gap time of 5 s, and ultrasonic power of 150 W). The mixture was filtered with a qualitative filter paper and 0.22 μm filter. The filtrate was used for the determination of soluble sugars. The separations were performed on a Dionex ICS3000 Multifunctional Ion Chromatograph (Dionex, Sunnyvale, CA, USA), using a Dionex CarboPac PA1 (2 × 250 mm) column at 30 °C with 40 mmol·L^−1^ NaOH as the eluent at a flow rate of 0.25 mL·min^−1^.

### 4.4. Volatiles Analysis

Headspace volatile production of banana fruit was determined using the SPME technique, as described previously [48] with minor modifications. Five grams of pulp from three fruit per replicate were homogenized and immediately introduced into a sealed vial. A 50/30 μm thick DVB/CAR/PDMS coated fiber (Supelco, Sigma-Aldrich, Bellefonte, PA, USA) was inserted into the vial containing the banana homogenate at a distance of 1.5 cm from the sample surface. After extraction for 25 min at room temperature, the fiber was inserted into the GC-MS (Finnigan Trace GC-MS, Santa Rosa, CA, USA). The injection port temperature was 250 °C, and the fiber was desorbed for 5 min (splitless). Separation was achieved on a DB-5MS (30 m × 0.25 mm × 0.1 µm) capillary column. The temperature program was as follows: 40 °C for 1 min, then increase to 60 °C at a rate of 2 °C·min^−1^, hold at 60 °C for 2 min, increase to 180 °C at 20 °C·min^−1^, and hold at 180 °C for 1 min. Helium was used as the carrier gas, and the flow rate was 1.0 mL·min^−1^. The FID temperature was 220 °C. The mass spectra (electronic impact (EI) 70 eV, quadrupole filter) were collected from *m*/*z* 35 to 300, generating 5.27 scans/s.

The compounds were identified by comparing the results obtained with reference mass spectra from the NIST02 library using the criterion of at least 75% similarity for the mass spectra, provided with the GC-MS software. The results were confirmed by using several pure components (butanoic acid, 3-methyl-, 3-methylbutyl ester, 1-butanol, 3-methyl-, acetate, acetic acid, hexyl ester, hexanal, 1-hexanol). The total volatile production was estimated by the sum of all peak areas identified in the chromatogram. The relative content percentage of each compound was calculated via the integrated peak area.

### 4.5. Statistical Analysis

Experiments were conducted using a completely randomized design. Each experiment contained at least three biological replicates. The data were analyzed using SPSS17.0 (Systat Software, SPSS17.0, San Jose, CA, USA). Figures were mainly plotted by SigmaPlot 10.0 (Systat Software, SigmaPlot 10.0, San Jose, CA, USA). Duncan’s multiple range test was used to determine the significant differences between treatment groups (*p* < 0.05). The results are expressed as mean ± S.E. Principal component analysis (PCA) was carried out using R software (https://www.r-project.org/, 27.06.2018) in order to provide a global overview of the volatiles profiles of Fenjiao and Brazilian fruit.

## Figures and Tables

**Table 1:** Changes in soluble saccharide content in banana fruit at various stages of ripening (mg·g^−1^).

Maturity	Glucose		Fructose		Sucrose	
	Fenjiao	Brazilian	Fenjiao	Brazilian	Fenjiao	Brazilian
Green	29.2 ± 14.1 ^a^	0.5 ± 0.1 ^b^	33.3 ± 11.3 ^a^	0.2 ± 0 ^b^	19.2 ± 4.7 ^a^	1.1 ± 0.3 ^b^
Turning	87.4 ± 3.3 ^a^	33.4 ± 7.3 ^b^	75.8 ± 2.9 ^a^	25.5 ± 5.6 ^b^	64.2 ± 8.5 ^a^	61.3 ± 20.5 ^a^
Full-Ripening	112.5 ± 2.5 ^a^	74.5 ± 4.1 ^b^	99.3 ± 4.7 ^a^	57.1 ± 3.5 ^b^	23.6 ± 5.0 ^b^	75.9 ± 16.7 ^a^

Note: Statistical analysis was performed by Duncan’s multiple range test. Means with different letters (^a,b^) within a row are significantly different at *p* < 0.05.

**Table 2:** Changes in volatiles compounds content in Brazilian banana fruit at various stages of ripening.

		Relative Content (%) ^1^		
Categories	Compounds	Green	Turning	Full-Ripening
Esters				
1	Pentyl butanoate	0.26 ^b^	--	3.05 ^a^
2	Ethyl acetate	--	0.22 ^b^	2.74 ^a^
3	2-Methylpentyl acetate	--	0.32 ^a^	0.13 ^b^
4	Isobutyl isobutyrate	--	0.21 ^b^	0.31 ^a^
5	Pentyl 3-methyl butanoate	--	0.30 ^b^	0.48 ^a^
6	Pentanoic acid, 3-methyl butanoate	--	0.45 ^a^	--
7	Hexyl butyrate	--	0.28 ^b^	0.49 ^a^
8	Butanoic acid, 4-hexen-1yl ester	--	0.15 ^b^	0.34 ^a^
9	Butanoic acid, 2-methyl cyclohexyl ester	--	0.15 ^a^	--
10	Pentanoic acid, 4-hexen-1yl ester	--	0.68 ^a^	--
11	Butanoic acid, 2-ethenyl hexyl ester	--	0.05 ^a^	--
12	Acetic acid, 1,4,-dimethylpent-4-enyl ester	--	0.48 ^a^	--
13	Isoamyl-2-methyl butyrate	--	0.30 ^b^	0.45 ^a^
14	Acetic acid, 2-methyl propyl ester	--	3.13 ^a^	3.09 ^a^
15	2-Pentanol, acetate	--	4.35 ^a^	4.17 ^a^
16	1-Butanol, 3-methyl-, acetate	--	14.34 ^a^	11.55 ^a^
17	Butanoic acid, butyl ester	--	1.62 ^a^	2.08 ^a^
18	Isobutyl isovalerate	--	2.73 ^a^	1.63 ^b^
19	Butanoic acid, 3-methylbutyl ester	--	18.7 ^a^	11.9 ^b^
20	1-methyl-hexyl acetate	--	6.78 ^a^	5.3 ^b^
21	3-methyl-butyl butyrate	--	0.73 ^a^	0.71 ^a^
22	Butanoic acid, 2-methylpropyl ester	--	--	3.51 ^a^
23	Butanoic acid, 3-methyl-, 3-methylbutyl ester	--	14.03 ^a^	13.71 ^a^
24	Butanoic acid, 1-methyl hexyl ester	--	1.61 ^a^	0.08 ^b^
25	Butanoic acid, 3-methyl hexyl ester	--	0.71 ^a^	0.74 ^a^
26	Propanoic acid, 3-methyl-, hexyl ester	--	--	0.05 ^a^
27	Isobutyl hexanoate	--	--	0.10 ^a^
28	Isopentyl hexanoate	--	--	1.04 ^a^
29	Ethyl-3-acetoxy hexanoate	--	--	0.17 ^a^
30	Butanoic acid, ethyl ester	--	--	2.06 ^a^
31	Acetic acid, butyl ester	--	--	1.31 ^a^
32	(*Z*)-3-Hexen-1-ol, acetate	--	--	1.05 ^a^
33	Cyclohexanol, 2-methyl-, acetate, (1*R*-*trans*)	--	--	1.04 ^a^
34	Propyl butyrate	--	--	0.03 ^a^
35	Butanoic acid, 1-methyl hexyl ester	--	--	0.83 ^a^
Total		0.26 ^b^	72.32 ^a^	74.1 ^a^
Alcohols				
36	1-Hexanol	21.47 ^a^	--	--
37	1-Butanol, 3-methyl	--	0.22 ^b^	0.35 ^a^
38	5-Octen-1-ol	--	0.05 ^a^	--
39	2-Pentanol	--	--	1.64 ^a^
40	Ethanol	--	--	0.92 ^a^
41	1-Pentanol, 2-methyl	--	--	3.63 ^a^
42	2-Heptanol	--	--	0.31 ^a^
Total		21.47 ^a^	0.27 ^c^	6.85 ^b^
Aldehydes				
43	(*Z*)-2-Heptenal	0.38 ^a^	--	--
44	Nonanal	0.30 ^a^	--	--
45	Hexanal	35.92 ^a^	2.03 ^b^	--
46	2-Hexenal	0.76 ^a^	--	--
47	*Trans*-2-hexenal	31.17 ^a^	8.34 ^b^	1.52 ^c^
48	*Trans*-2-*cls*-6-nonadienl	0.15 ^a^	--	--
49	(*E*)-2-Nonenal	0.63 ^a^	--	--
Total		69.31 ^a^	51.12 ^a^	4.44 ^b^
Ketones				
50	2-Pentanone	--	0.58 ^b^	1.07 ^a^
51	2-Undecanone	--	0.04 ^a^	--
52	Heptanone	--	--	0.51 ^a^
53	2-Heptanone	--	--	0.51 ^a^
54	5-Hepten-2-one	--	--	0.08 ^a^
Total		--	0.62 ^b^	2.17 ^a^
Acids				
55	(*Z*)-3-Octen-1-ol, acetate	--	0.23 ^a^	--
56	4-Hexen-1-ol, acetate	--	0.24 ^a^	--
57	*Trans*-3-octen-1-ol, acetate	--	--	0.11 ^a^
Total		--	0.47 ^a^	0.11 ^a^
Hydrocarbons				
58	Limonene	0.18 ^a^	--	--
59	2-octyne	--	0.25 ^a^	--
60	Cyclobutane	--	0.18 ^a^	--
61	(*Z*,*Z*)-1,4-Cyclooctadiene	--	0.04 ^a^	--
62	Bicyclo [10.1.0] tridecane	--	0.79 ^a^	--
Total		0.18 ^b^	1.26 ^a^	--
Phenols				
63	Eugenol	--	--	0.17 ^a^
Total		--	--	0.17 ^a^
Others		8.78 ^b^	14.69 ^a^	15.04 ^a^

--: not detectable. ^1^ Statistical analysis was performed by Duncan’s multiple range test. Means with different letters (^a,b,c^) within a row are significantly different at *p* < 0.05.

**Table 3:** Changes in volatiles compounds content in Fenjiao banana fruit at various stages of ripening.

		Relative Content (%) ^1^		
Categories	Compounds	Green	Turning	Full-Ripening
Esters				
1	Ethyl acetate	--	0.29 ^b^	2.78 ^a^
2	1-Butanol, 3-methyl-, acetate	--	--	21.29 ^a^
3	Hexanoic acid, ethyl ester	--	0.18 ^b^	1.52 ^a^
4	4-Hexen-1-ol, acetate, (*Z*)	--	--	0.84 ^a^
5	1-methyl hexyl acetate	--	--	0.41 ^a^
6	Butanoic acid, 3-methyl-, butyl ester	--	--	0.30 ^a^
7	Isoamyl-2-methyl butyrate	--	--	1.88 ^a^
8	Ethyl-3-hydroxy hexanoate	--	--	0.40 ^a^
9	3-Methylbutyl decanoate	--	--	0.77 ^a^
10	*Trans*-2-nonenoate	0.18 ^a^	0.04 ^a^	--
11	Hexyl hexanoate	0.15 ^b^	1.52 ^a^	--
12	Hexanoic acid, 3-hexenyl ester, (*Z*)	0.15 ^a^	0.09 ^a^	--
13	Pentanoic acid, pentyl ester	--	--	1.04 ^a^
14	Diisobutyl phthalate	--	0.08 ^a^	--
15	Butanoic acid, 3-methyl-, 2-methyl propyl ester	--	--	0.38 ^a^
16	Isobutyl acetate	--	--	0.91 ^a^
17	Butyl acetate	--	--	1.66 ^a^
18	Butanoic acid, 3-methyl-, 3-methylbutyl ester	--	--	6.24 ^a^
19	Butanoic acid, 4-hexen-1yl ester	--	--	0.61 ^a^
20	Butanoic acid, 2-methylpropyl ester	--	--	3.43 ^a^
21	Butanoic acid, 3-methylbutyl ester	--	0.87 ^a^	--
22	Butanoic acid, butyl ester	--	--	2.87 ^a^
23	Butanoic acid, ethyl ester	--	--	1.46 ^a^
24	Butanoic acid, amyl ester	--	--	0.21 ^a^
25	Hexanoic acid, 3-methylbutyl ester	--	1.01 ^a^	--
26	Propanoic acid, 2-methylbutyl ester	--	--	0.82 ^a^
27	Hexanoic acid, butyl ester	--	--	0.54 ^a^
28	Octanoic acid, 3-methyl butyl ester	--	--	2.67 ^a^
29	Propanoic acid, 3-methyl, 3-methyl butyl ester	--	--	5.71 ^a^
30	2-(1-Pentyloxy)-ethyl acetate	--	--	0.37 ^a^
31	1-Methyl butyl acetate	--	--	3.00 ^a^
32	Acetic acid, hexyl ester	0.20 ^a^	1.30 ^a^	6.11 ^a^
33	(*Z*)-3-Hexen-1-ol acetate	--	0.18 ^a^	--
34	Butanoic acid, 1-methyl, hexyl ester	--	--	21.79 ^a^
Total		0.68 ^c^	5.56 ^b^	90.01 ^a^
Alcohols				
35	Menthol	--	0.02 ^a^	--
36	1-Butanol, 3-methyl	--	0.60 ^b^	1.45 ^a^
37	1-Hexanol	21.12 ^a^	12.33 ^b^	--
38	1-Nonanol	--	14.65 ^a^	--
39	3-Pentanol, 2,4-dimethyl	--	--	1.00 ^a^
40	1-Hexanol, 5-methyl	--	12.35 ^a^	--
Total		21.12 ^b^	39.95 ^a^	2.45 ^c^
Aldehydes				
41	Nonanal	--	0.15 ^a^	--
42	Dodecanal	0.06 ^a^	0.02 ^a^	--
43	Hexanal	22.5 ^a^	17.25 ^a^	--
44	2-Hexenal	11.16 ^a^	10.5 ^a^	--
45	*Trans*-2-hexenal	25.08 ^a^	23.13 ^a^	4.44 ^b^
46	Decanal	--	0.07 ^a^	--
Total		58.8 ^a^	51.12 ^a^	4.44 ^b^
Ketones				
47	2-Pentanone	--	--	0.61 ^a^
Total		--	--	0.61 ^a^
Acids				
48	Hexadecenoic acid, Z-11-	--	--	1.26 ^a^
49	Tetradecanoic acid	--	--	0.88 ^a^
Total		--	--	2.04 ^a^
Hydrocarbons				
50	Dipentene	0.30 ^a^	--	--
51	Nonadecane	0.07 ^a^	--	--
52	*n*-Pentadecane	0.17 ^a^	0.1 ^a^	--
53	Hexadecane	0.09 ^a^	0.8 ^a^	--
54	Dodecane	0.24 ^a^	0.08 ^a^	--
55	Eicosane	0.15 ^a^	--	--
56	Tetradecane	0.20 ^a^	--	--
57	Docosane	--	0.07 ^a^	--
58	Tridecane	--	0.02 ^a^	--
Total		1.22 ^a^	1.07 ^a^	--
Phenols				
59	2,6-Di-*tert*-butyl-4-methylphenol	0.81 ^a^	0.12 ^b^	--
Total		0.81 ^a^	0.12 ^b^	--
Others		17.37 ^a^	2.18 ^b^	0.45 ^b^

--: not detectable. ^1^ Statistical analysis was performed by Duncan’s multiple range test. Means with different letters (^a,b,c^) within a row are significantly different at *p* < 0.05.

**Table 4:** Comparison of the main volatiles compounds in Brazilian and Fenjiao banana fruit at various stages of ripening.

	Relative Content (%)	Green		Turning		Full-Ripening ^1^	
Compounds		Fenjiao	Brazilian	Fenjiao	Brazilian	Fenjiao	Brazilian
Total (Esters)		0.68 ^c^	0.26 ^c^	5.56 ^b^	72.32 ^a^	90.01 ^a^	74.14 ^a^
Acetate		0.20 ^d^	--	1.77 ^c^	18.17 ^b^	32.75 ^a^	18.69 ^b^
Methyl-acetate		--	--	--	7.10 ^a^	3.41 ^b^	6.48 ^a^
Propionate		--	--	--	--	--	--
Methylpropionate		--	--	--	--	6.53 ^a^	0.05 ^b^
Butyrate		--	0.26 ^d^	0.87 ^d^	20.95 ^c^	30.37 ^a^	23.46 ^b,c^
Methylbutyrate		--	--	--	20.62 ^a^	8.8 ^b^	18.94 ^a^
Pentanoate		--	--	--	1.13 ^a^	1.04 ^a^	--
Hexanoate		0.30 ^c^	--	2.80 ^a^	--	2.46 ^a^	1.14 ^b^
Octoate		--	--	--	--	2.67 ^a^	--
Decanoate		--	--	--	--	0.77 ^a^	--
Others		0.18 ^c^	--	0.12 ^c^	4.35 ^a^	1.21 ^b^	5.38 ^a^
Total (Aldehydes)		58.50 ^a,b^	69.31 ^a^	51.12 ^b^	10.37 ^c^	4.44 ^d^	1.52 ^e^
Hexanal		22.50 ^b^	35.92 ^a^	17.25 ^b^	2.03 ^c^	--	--
2-Hexenal		11.16 ^a^	0.76 ^c^	10.5 ^b^	--	--	--
(*E*)-2-Hexenal		25.08 ^a,b^	31.17 ^a^	23.13 ^b^	8.34 ^c^	4.44 ^d^	1.52 ^e^
Others		0.06 ^b^	1.46 ^a^	0.22 ^b^	--	--	--
Total (Alcohols)		21.12 ^b^	21.47 ^b^	39.95 ^a^	0.27 ^e^	2.45 ^d^	6.85 ^c^
1-Hexanol		21.12a	21.47 ^a^	12.33 ^b^	--	--	--
Others		--	--	27.62 ^a^	0.27 ^d^	2.45 ^c^	6.85 ^b^

--: not detectable. ^1^ Statistical analysis was performed by Duncan’s multiple range test. Means with different letters (^a,b,c,d,e^) within a row are significantly different at *p* < 0.05.

## Supplementary Materials

The Supplementary Materials are available online.
