# Supplementary material for: Comparative Study of Volatile Compounds in the Fruit of Two Banana Cultivars at Different Ripening Stages
Source: Molecules. 2018 Sep 25;23(10):2456. doi: 10.3390/molecules23102456 (PMC6222428; doi:10.3390/molecules23102456)
Supplement: Supplementary file 1 [file molecules-23-02456-s001.zip › Supplementary materials/Supplementary Figure 1.pdf]

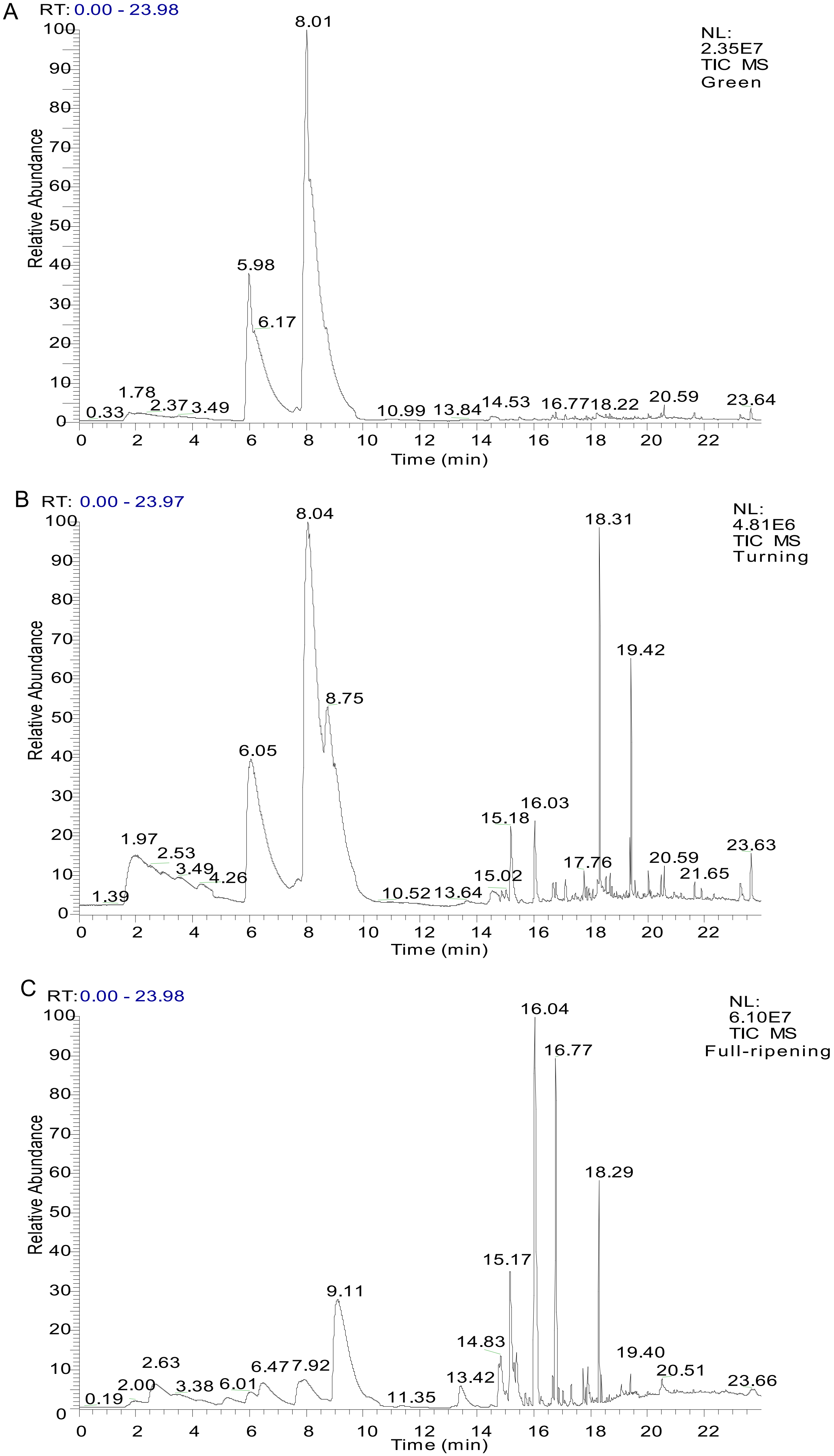
A
RT: 0.00 - 23.98
NL: 2.35E7 TIC MS Green
Relative Abundance
Time (min)
8.01
5.98
6.17
1.78
2.37
3.49
0.33
10.99
13.84
14.53
16.77
18.22
20.59
23.64
B
RT: 0.00 - 23.97
NL: 4.81E6 TIC MS Turning
8.04
18.31
19.42
8.75
6.05
16.03
15.18
1.97
2.53
3.49
4.26
1.39
10.52
13.64
15.02
17.76
20.59
21.65
23.63
C
RT: 0.00 - 23.98
NL: 6.10E7 TIC MS Full-ripening
16.04
16.77
18.29
15.17
9.11
14.83
2.63
2.00
3.38
6.01
6.47
7.92
0.19
11.35
13.42
19.40
20.51
23.66

Supplementary Fig. 1. Aroma volatile production of Fenjiao banana at the ripening stage of green (A), turning(B) and full-ripening(C) identified by GC-MS.
