# Supplementary material for: Comparative Study of Volatile Compounds in the Fruit of Two Banana Cultivars at Different Ripening Stages
Source: Molecules. 2018 Sep 25;23(10):2456. doi: 10.3390/molecules23102456 (PMC6222428; doi:10.3390/molecules23102456)
Supplement: Supplementary file 1 [file molecules-23-02456-s001.zip › Supplementary materials/Supplementary Figure 2.pdf]

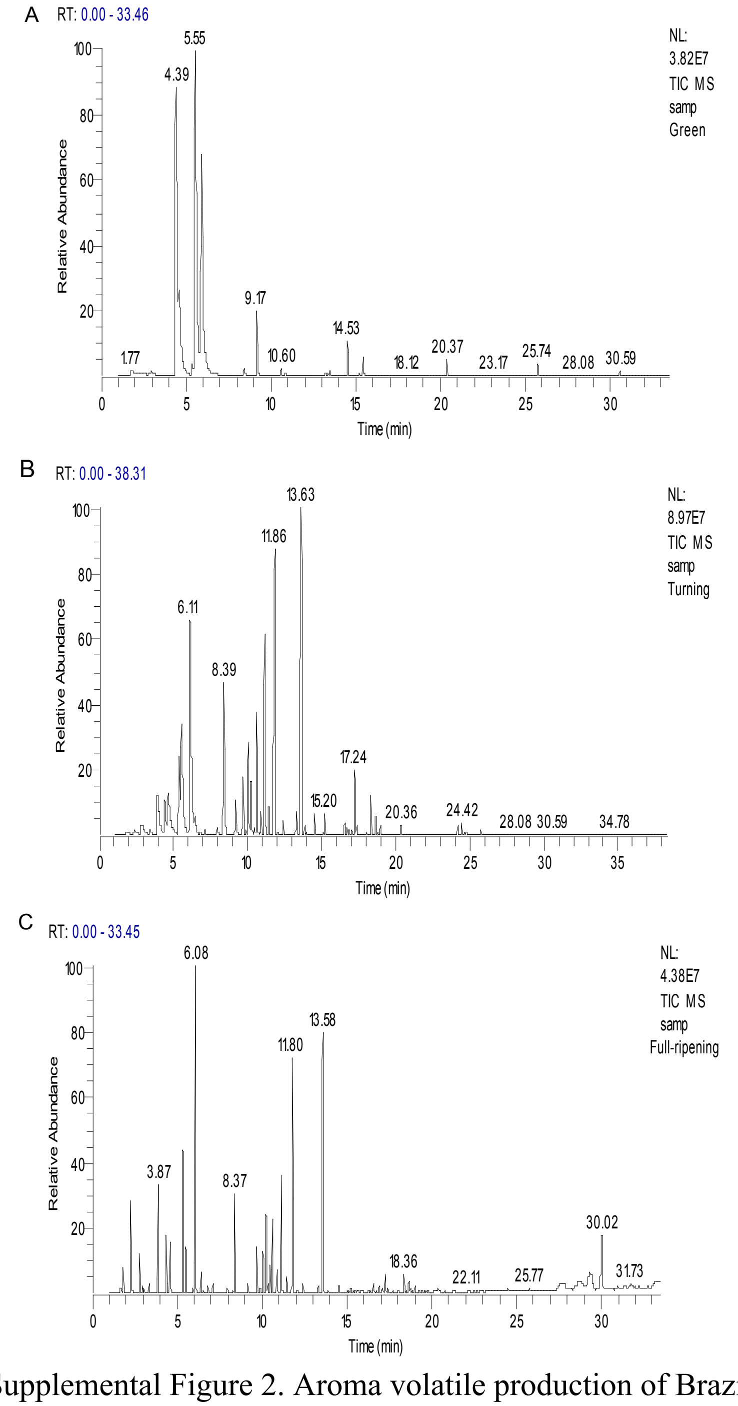

Supplemental Figure 2. Aroma volatile production of Brazilian banana at the ripening stage of green (A), turning(B) and full-ripening(C) identified by GC-MS.

Supplementary Fig. 2. Aroma volatile production of Brazilian banana at the ripening stage of green (A), turning(B) and full-ripening(C) identified by GC-MS.
